# Underwater endoscopic submucosal dissection for recurrent superficial esophageal cancer at the site of stenosis after endoscopic resection

**DOI:** 10.1055/a-2598-3672

**Published:** 2025-05-28

**Authors:** Noriaki Ito, Shunsuke Yoshii, Tomoki Michida, Ryu Ishihara

**Affiliations:** 1 53312 Department of Gastrointestinal Oncology, Osaka International Cancer Institute, Osaka, Japan

Repeated endoscopic submucosal dissection (ESD) for local recurrence after endoscopic resection can be challenging due to diffuse submucosal fibrosis. In addition, extensive esophageal ESD can lead to luminal stenosis, and ESD for recurrent lesions in the stenosis is complicated because of severe submucosal fibrosis and poor maneuverability 1 2 . Herein, we present two cases of recurrent esophageal cancer in areas of stenosis caused by prior extensive ESD, which were successfully resected using underwater ESD with a tapered tip hood ( Video 1 ).

Underwater endoscopic submucosal dissection for recurrent superficial esophageal cancer at the site of stenosis after endoscopic resection.Video 1

The first case involved a 67-year-old man with an 8-mm superficial adenocarcinoma in the middle thoracic esophagus. The lesion was located inside the scar of a previous semi-circumferential ESD for long-segment Barrett’s esophageal adenocarcinoma. The second case involved an 84-year-old man with a 12-mm squamous cell carcinoma in the middle thoracic esophagus. The lesion was also located on the scar from a previous ESD and ablation therapy. These lesions were located at the stenosis site before ESD ( Fig. 1 ). Underwater ESD was performed using a tapered tip hood (CAST hood; TOP Corporation, Tokyo, Japan). The 1.3 times magnified endoscopic view 3 and the clear underwater visibility allowed precise dissection of the fibrotic area. The tapered design of the hood prevented collisions with the esophageal wall ( Fig. 2 , Fig. 3 ). Consequently, we achieved curative en bloc resection of the lesions without adverse events.

**Fig. 1:**
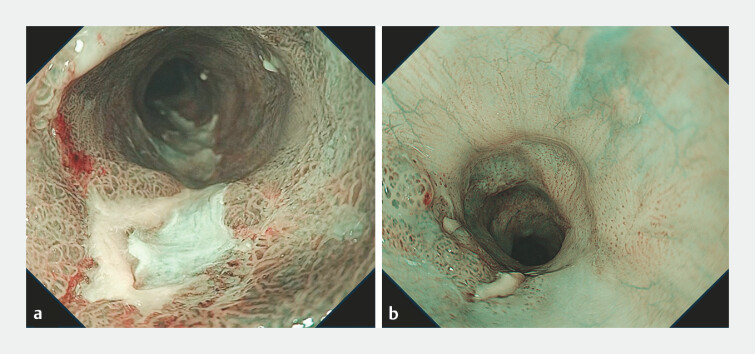
The target lesions located at the stenosis site of the previous endoscopic submucosal dissection. **a** The endoscopic view with narrow-band imaging of the lesion in case 1. **b** The endoscopic view with narrow-band imaging of the lesion in case 2.

**Fig. 2:**
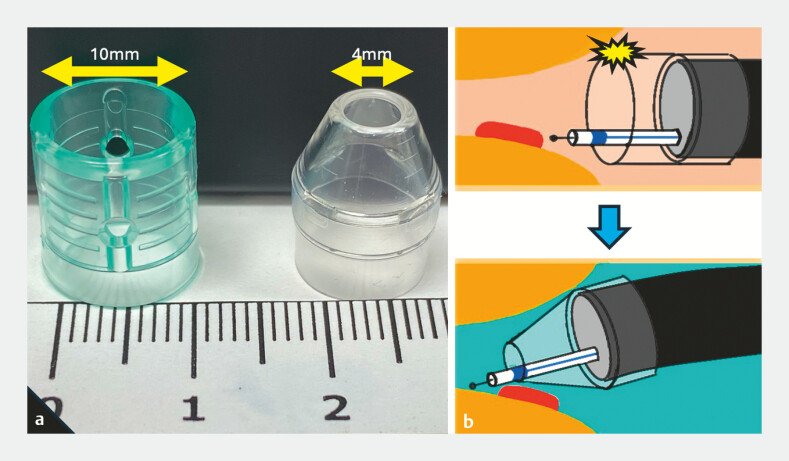
The difference between a non-tapered hood and a tapered tip hood. **a** Comparison of the non-tapered hood (Elastic Touch; TOP Corporation, Tokyo, Japan) and tapered tip hood. **b** Scheme of underwater endoscopic submucosal dissection with the tapered tip hood. The anal side of the stenosis can be accessed without colliding with the surrounding walls.

**Fig. 3:**
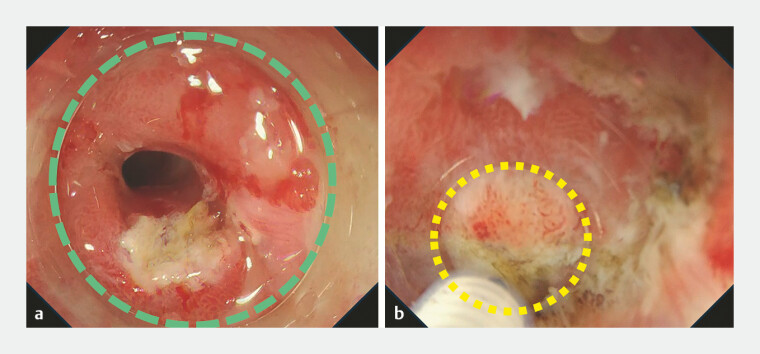
Endoscopic views during endoscopic submucosal dissection in case 1. **a** Endoscopic view with a non-tapered hood. The tip of the hood collides with the surrounding walls (green dotted circle). **b** Underwater endoscopic view with a tapered tip hood. The yellow dotted circle indicates the opening of the hood. We approached the anal side of the lesion without colliding with the surrounding walls.

ESD for superficial esophageal cancers has demonstrated an excellent R0 resection rate regardless of tumor size and circumference; however, post-ESD stenosis remains an issue. Moreover, the high annual incidence of metachronous cancers after esophageal ESD occasionally results in recurrent lesions at the site of stenosis. This report suggests that underwater ESD with a tapered tip hood is a beneficial approach due to clear visibility, improved reachability, and maneuverability of the technique at the stenosis site, allowing safe and effective ESD.

Endoscopy_UCTN_Code_TTT_1AO_2AG_3AD

## Notes

Endoscopy E-Videos https://eref.thieme.de/e-videos
*E-Videos* is an open access online section of the journal *Endoscopy* , reporting on interesting cases and new techniques in gastroenterological endoscopy. All papers include a high-quality video and are published with a Creative Commons CC-BY license. Endoscopy E-Videos qualify for HINARI discounts and waivers and eligibility is automatically checked during the submission process. We grant 100% waivers to articles whose corresponding authors are based in Group A countries and 50% waivers to those who are based in Group B countries as classified by Research4Life (see: https://www.research4life.org/access/eligibility/ ). This section has its own submission website at https://mc.manuscriptcentral.com/e-videos .

## References

[1] Ono S Fujishiro M Niimi K Predictors of postoperative stricture after esophageal endoscopic submucosal dissection for superficial squamous cell neoplasms Endoscopy 2009 41 661 665 10.1055/s-0029-1214867 19565442

[2] Zhang J Wang H Shi M Salvage endoscopic submucosal resection for residual esophageal superficial cancer involving a stenotic anastomosis: a challenging but desirable indication Endoscopy 2023 55 E908 E909 10.1055/a-2106-1096 37442172 PMC10344609

[3] Nomura T Sugimoto S Oyamada J GI endoscopic submucosal dissection using a calibrated, small-caliber-tip, transparent hood for lesions with fibrosis VideoGIE 2021 6 301 304 34278091 10.1016/j.vgie.2021.03.001 PMC8267961

